# Development and validation of nomograms including individual- and area-level variables to predict risk of fatal and non-fatal cardiovascular diseases among Russian population

**DOI:** 10.1371/journal.pone.0324736

**Published:** 2025-06-02

**Authors:** Anastasia A. Zelenina, Svetlana A. Shalnova, Oksana M. Drapkina

**Affiliations:** 1 Department of Epidemiology of Chronic Non-Communicable Diseases, National Medical Research Center for Therapy and Preventive Medicine of the Ministry of Healthcare of the Russian Federation, Moscow, Russia; 2 Department of Fundamental and Applied Aspects of Obesity, National Medical Research Center for Therapy and Preventive Medicine of the Ministry of Healthcare of the Russian Federation, Moscow, Russia; University of Montenegro-Faculty of Medicine, MONTENEGRO

## Abstract

**Introduction:**

Cardiovascular diseases (CVD) are the greatest threat to health worldwide and in Russia. Our study aimed to use Cox proportional hazards models to develop cardio-vascular risk scores and nomograms based on prospective data from studies conducted in Russia.

**Methods:**

All materials used in this study were obtained from the epidemiological study “Epidemiology of Cardiovascular Diseases in the Regions of the Russian Federation” (ESSE-RF): ESSE-RF (2012-2014) and ESSE-RF2 (2017). A total of 18,454 individuals without CVD aged 25–64 years were included in our study. The participants were randomly divided into a training and testing set at a ratio of 7:3. The Russian deprivation index and its components (social, economic and environmental) were used as area-level predictors. To select the best potential predictive variables for our models, the random forests variable selection algorithm based on minimal depth was used. To predict three- and five-year CVD-free survival, four prognostic nomograms were developed from the results of multivariate analysis.

**Results:**

The nomograms had considerable discriminative power, calibrating abilities and clinical effectiveness. The time dependent AUC was > 0.7 for the prediction of CVD-free survival in both the training and testing sets.

**Conclusion:**

For the first time, the nomograms have been created that include area-level predictors (socio-economic and environmental) and lipid spectrum indicators (triglycerides, high-density lipoprotein cholesterol and low-density lipoprotein cholesterol) and assess the probability of fatal and non-fatal cardiovascular events among the Russian population.

## Introduction

Cardiovascular diseases (CVD) are the greatest threat to health worldwide and in Russia. In 2021, 20.5 million people died from a cardiovascular condition globally (around one-third of all global deaths) [1]. According to World Health Organization, in 2019 the top two causes of death were ischemic heart disease (385 deaths per 100,000 population) and stroke (224 deaths per 100,000 population) in Russia [2].

Undoubtedly, the most effective measure to combat cardiovascular disease is the early identification of risk factors. A number of large studies have made significant contributions to the understanding of individual behavioral and biological risk factors, for example the Framingham Study [3] and the North Karelia Project [4].

During the Framingham Study, the concept of risk factors for the development of CVD was first introduced and the theory of modified and unmodified risk factors was put forward. The study also identified risk factors for CVD, such as smoking, increased levels of low-density lipoprotein cholesterol (LDL-C), a diet high in cholesterol/animal fat, arterial hypertension, diabetes mellitus (DM), insufficient physical activity, obesity, postmenopausal status, etc. All these factors had a direct impact on the occurrence and development the disease.

Certainly, studying the influence of risk factors of the so-called direct exposure has been productive. Thus, during the implementation of the “North Karelia” project, the main attention was paid to general lifestyle changes (especially nutrition and smoking), which, when implemented as a preventive intervention, led to a decrease in mortality from coronary heart disease (CHD) by 56% in men and by 64% in women aged 35–64 years. However, the reduction in the level of risk factors explained only 53% of the total reduction in mortality from CHD, 23% of the total decline in CHD was due to treatment, and about 24% of the total decline in incidence was not explained by the risk factors included in the model. The data obtained suggested that in addition to direct impact factors, there are factors that indirectly affect health. Awareness of this fact served as an impetus for the development of concepts of social determinants of health (SDOH).

The US Centers for Disease Control and Prevention provides the following definition of SDOH: “the nonmedical factors that influence health outcomes. They are the conditions in which people are born, grow, work, live, and age. These forces and systems include a wide set of forces and systems that shape daily life such as economic policies and systems, development agendas, social norms, social policies, and political systems” [5].

The literature is replete with studies pertaining to socio-economic and environmental inequities in healthcare [6–11]. Studying the impact of social determinants on population health, they adhere to a deprivation approach, which defines inequalities in socio-economic and environmental conditions and health [12].

In the studies, individual and area-level indicators of deprivation can be identified. For example, few studies [13,14] showed the association between self‐reported indicators of deprivation and risk of atherosclerotic cardiovascular diseases (ASCVD).

Area-level indicators, in turn, are presented in studies as separate indicators [14] or as part of the index of deprivation [15].

Area-level deprivation indicators improve cardiovascular risk assessment tools in case of spatial differences in cardio-vascular health [16,17]. The use of a unified national CVD risk assessment model in such conditions will certainly lead to an inadequate assessment of risk: underestimation of risk in some environmental conditions (regions) and overestimation in others. The use of index to measure the area-level deprivation also has its advantages, as it allows one to take into account more complex mechanisms of interaction between social, economic and environmental indicators when studying their impact on health. In world scientific practice, awareness of these facts has already led to some national cardiovascular risk scores began to include environmental characteristics of residence within individual countries. For example, the New Zealand PREDICT score includes as a risk factor – New Zealand index of socioeconomic deprivation [18], the British QRISK score – Townsend deprivation index [19], the Scottish ASSIGN score – the Scottish Index of Multiple Deprivation [20].

A number of studies confirm the association of these deprivation indices with cardiovascular disorders [21–27].

In addition to cardiovascular risk score, deprivation indices are included in tools risk assessment of type 2 diabetes [28], common cancers [29], survival in patients with colorectal cancer [30], breast cancer mortality [31] lung cancer [32], oesophageal cancer [33], ischemic stroke [34], dementia [35], and asthma exacerbations [36].

In Russian medical practice used a tool for assessing fatal risk of CVD – SCORE [37] and its updated version, a tool for assessing fatal and non-fatal risk of CVD – SCORE2 [38]. Both of these tools were validated for the Russian population and include only traditional risk factors. Unfortunately, as far as we know, there is no cardiovascular risk score for the Russian population, which, in addition to the traditional risk factors, includes characteristics of areas of residence. Therefore, cardio-vascular risk score included area-level social determinants is needed for Russian adults.

Our study aimed to use Cox proportional hazards models to develop cardio-vascular risk scores and nomograms [39] based on prospective data from studies conducted in Russia. The models’ discrimination, clinical utility, calibration and internal validation will all be assessed.

## Materials and methods

### Data sources

All materials used in this study were obtained from the epidemiological study “Epidemiology of Cardiovascular Diseases in the Regions of the Russian Federation” (ESSE-RF): ESSE-RF and ESSE-RF2. The recruitment period for ESSE-RF began on September 15, 2012 and ended on June 10, 2014. The recruitment period for ESSE-RF2 was from July 3, 2017, to February 14, 2018. The ESSE-RF was registered at ClinicalTrials.gov as NCT02449460. A total of 28,817 individuals aged 25–64 years were examined. The studies were performed using a single protocol. The samples were drawn based on the Kish method [40], which provides for systematic, multi-step, random community-based sampling on the premises of medical and preventive treatment facilities. The ESSE-RF and ESSE-RF2 studies have been carried out in two phases: 1st phase – the examination of study participants to investigate CVD risk factors and 2nd – prospective observation of the vital status of the cohort examination and assessment of causes of death. Prospective monitoring the occurrence of fatal and non-fatal events are held annually, starting from next year after completion of the first phase of the studies.

Both databases disclose participant demographic information, laboratory and instrumental indicators, and survival status. Detailed data collection methods have been previously published [41,42].

The study was conducted in accordance with the Transparent Reporting of a Multivariable Prediction Model for Individual Prognosis or Diagnosis (TRIPOD) guidelines (S1 Table) [43].

### Ethics statement

The study protocol was approved by the following Ethics Committees: National Medical Research Center for Therapy and Preventive Medicine, Russian Cardiology Research-and-Production Complex, and Federal Almazov North-West Medical Research Centre. All study participants provided written informed consent before starting the survey and the interview.

### Exclusion criteria

**Fig 1:**
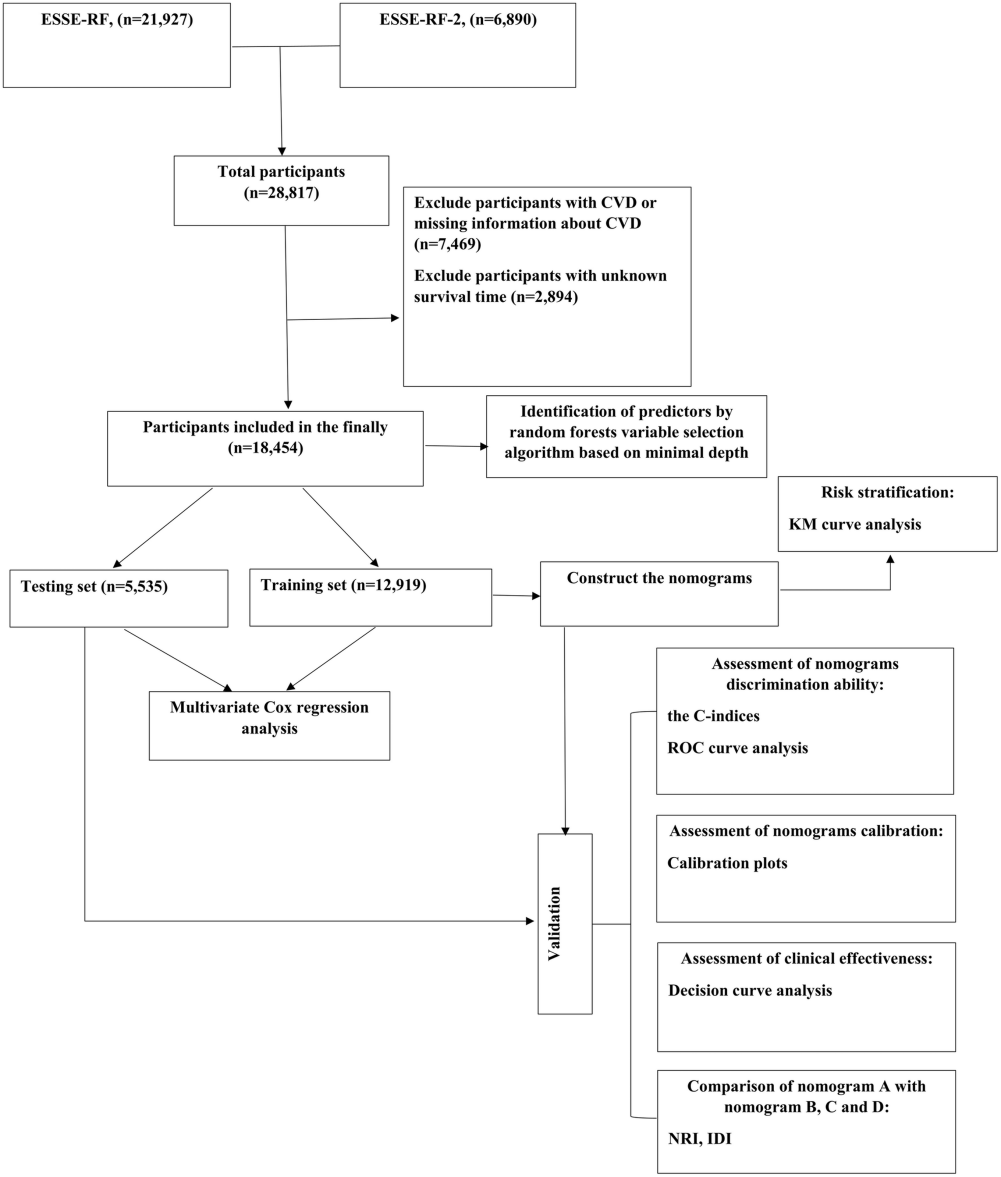
The flowchart of the study design. KM, Kaplan-Meier; IDI, integrated discrimination improvement; NRI, net reclassification improvement; ROC, receiver operating characteristic; CVD, cardiovascular diseases.

Exclusion criteria was applied during the data selection process. Participants who met any of the following criteria were excluded from the study: CVD or missing information about CVD at the 1st phase of the study and unknown survival time. A positive answer to the question: “Has a doctor ever told you that you have/had the following diseases: stroke (cerebral thrombosis or hemorrhage), myocardial infarction, CHD (angina), heart rhythm disturbances, other heart diseases?” was considered an identifier of a history of CVD. Fig 1 showed the workflow of this study.

### Individual-level independent variables

The analysis included sex, age, place of residence (rural/urban), smoking status (current/former/never), DM, body mass index (BMI), systolic blood pressure (SBP), heart rate (HR), the use of antihypertensive medications and laboratory parameters such as high-density lipoprotein cholesterol (HDL-C), LDL-C, triglycerides (TG), uric acid, creatinine and glucose.

Blood pressure and HR were measured using an automatic blood pressure monitor (Omron, Kyoto Japan) after patients were requested to rest for 5 minutes in a seated position.

The body height and weight were measured in participants with lightweight clothing and without shoes. BMI was calculated using the formula weight/height^2^. 

Information on the use of antihypertensive medication, smoking status, place of residence, and DM were self-reported by the participants. Respondents who smoked at least one cigarette/cigarette per day or who quit smoking less than a year ago were considered to be smokers. Three groups of people were identified according to their smoking status: current, former and never.

Blood sampling for biochemical testing were collected via venipuncture after 12 hours of fasting. Blood serum was obtained by low-speed centrifugation at 900 g for 20 minutes at a temperature of +4°С. Samples of biological material were frozen and stored at a temperature not exceeding minus 20°С. Transportation of biomaterials was carried out by specialized services. Lipid spectrum indicators (the levels of TG, LDL-C and HDL-C), as well as uric acid, creatinine and glucose, were determined on an Abbot Architect c8000 autoanalyzer using diagnostic kits from Abbot Diagnostic (USA). Standardization and quality control of the analysis were carried out in accordance with the requirements of the Federal System for External Quality Assessment of Clinical Laboratory Research.

### Missing data

Missing values in the dataset were: self-reported doctor-diagnosed DM (0.05%), use of antihypertensive medications (0.38%), SBP (0.29%), HR (0.3%), BMI (0.63%), HDL-C (1.52%), LDL-C (1.9%), creatinine (1.66%), blood glucose (1.53%), uric acid (1.57%), and smoking status (0.1%). Bootstrap-based Expectation-Maximization algorithm was employed to impute missing values using “Amelia” package [44].

### Definitions

Survival status: The outcome of a participant without CVD at the time of examination either event or censored.

Event: CVD death (based on the International Classification of Diseases, 10th revision (ICD-10) code recorded as the underlying cause of death) or CHD (non-fatal) or a cerebrovascular accident (non-fatal).

Survival time: Measure of the follow-up time from a defined starting point from the examination of participants at the 1st phase of the study to the occurrence of the event.

### Area-level deprivation index

Russian deprivation index (RDI) was used to measure level of deprivation. Indicators for the index were obtained from official statistical publications of the Federal State Statistics Service of Russia and the All-Russian Census of Population for 2010 [45]. Principal component analysis was used to create the index. RDI includes 17 indicators reflecting the socio-economic and environmental deprivation at the regional level in Russia (S2 and S3 Tables). The deprivation index and its components levels were divided into four quantiles. The deprivation effect was assessed by comparing four quantiles. The first quantile (Q1) is the least deprived area; the fourth quantile (Q4) is the most deprived area. The index measures general deprivation, and its components measure social, economic and environmental deprivation, respectively (S4–S7 Tables). Further details on the creating of RDI have been described in the previous publication [46].

### Statistical analysis

All statistical analyses were conducted using R software (version 4.4.1; https://www.R‐project.org) with the R base package. The participants were randomly divided into a training and testing set at a ratio of 7:3 using the “caret” package. The training set was used to develop the models and the testing set for internal validation.

Continuous variables were described by median (Med) and the interquartile range (IQR). Categorical variables were expressed as percentages (%) and absolute numbers (n). Variables between sets were assessed by Pearson’s Chi-squared test and Wilcoxon rank sum test as appropriate. Multicollinearity was evaluated by the variance inflation factor (VIF) using package “car”. VIF > 4.0 was interpreted as indicating multicollinearity [47].

To select the best potential predictive variables for our models, the random forests variable selection algorithm based on minimal depth from the package “randomForestSRC” was used [48]. The features selected in the data set using the random forests variable selection algorithm were utilized to construct nomograms through a multivariate Cox regression analysis to predict participants’ 3-year and 5-year survival probability. These variables were then taken into multivariate Cox regression models. For the current study we used clustered data [49]: individual-level variables (e.g., sex, age, smoking status, DM, etc.) and cluster-level variables (RDI and its components) thus Cox-Proportional hazards models with Huber-White cluster sandwich estimator of variance were used [50,51] To assess the impact of the prognostic factors for 3-year and 5-year CVD-free survival (fatal/non-fatal), Hazard Ratio (HR) and 95% confidence interval (CI) were calculated using multivariate Cox proportional risk regression models. Four Cox regression models were constructed. All models include the same laboratory and anthropometric indicators, the difference lies in area-level indicators. Model 1 includes an indicator of general deprivation, model 2 – social deprivation, model 3 – economic deprivation, model 4 – environmental deprivation. Then, to predict the survival probability of CVD (fatal/no-fatal), a nomogram models were constructed using “rms” package. Model 1 used to develop nomogram A, model 2 – nomogram B, model 3 – nomogram C, model 4 – nomogram D.

The reliability and accuracy of the nomograms were assessed via Harrell’s concordance-index (C-index), receiver operating characteristic (ROC) analysis and calibration curves. The area under the time-dependent ROC curve (AUC) was used to evaluate the prediction discrimination of the nomograms. In the regression model, AUC value was similar to C-index, and AUC value > 0.7 was considered to have better discrimination ability [52]. Calibration curves were plotted using the R software “rms” package to evaluate the calibration of the nomograms. To further estimate the clinical usefulness of the nomograms, decision curve analysis (DCA) was conducted using the “dcurves” package.

The integrated discrimination improvement (IDI) and net reclassification improvement (NRI) were used to calculate the performance improvement of the nomogram A compared to the nomograms B, C and D.

Additionally, the total score for all participants was computed utilizing these nomograms and two cut-off points for these total scores were determined using package “rolr” [53]. The participants were divided into the low-risk, moderate-risk and high-risk groups. To evaluate the prognostic differences between the three risk groups, the Kaplan-Meier (KM) method was used for survival analysis employing the log-rank test with Bonferroni correction. P < 0.05 for both sides was considered significant.

## Results

### Variable selection

VIF was assessed among the independent variables (sex, age, place of residence (rural/urban), smoking status (current/former/never), DM, BMI, SBP, HR, HDL-C, LDL-C, TG, uric acid, creatinine, glucose, use of antihypertensive medications, and values of RDI (its components)). All variables had VIF < 4.0 and were included in random forest variable selection analysis.

**Fig 2:**
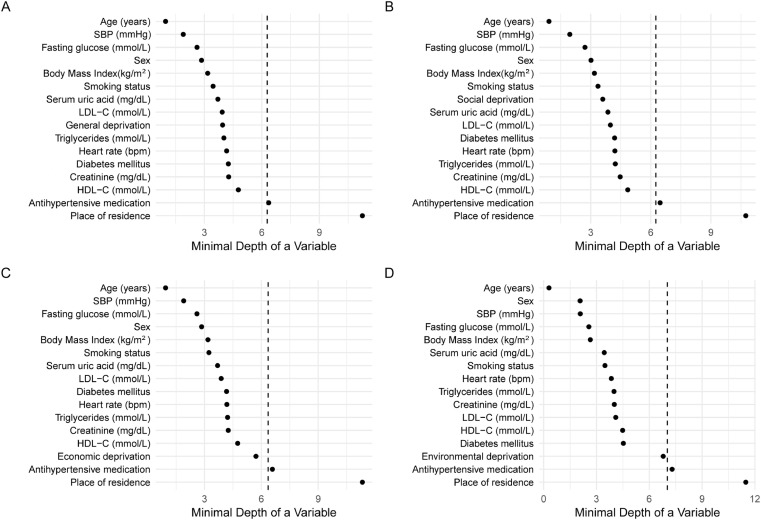
Minimal depth variables in rank order, most important at the top. Vertical dashed line indicates the maximal minimal depth for important variables. Indicators for nomogram A **(A)**, nomogram B **(B)**, nomogram C **(C)**, nomogram D **(D)**. SBP, systolic blood pressure; LDL-C, low-density lipoprotein cholesterol; HDL-C, high-density lipoprotein cholesterol.

The results of random forest selection process (the tree minimal depth approach) indicated the following independent variables: sex, age, smoking status (current/former/never), DM, BMI, SBP, HR, HDL-C, LDL-C, TG, uric acid, creatinine, glucose, and values of RDI (its components) as important variables and therefore they were included in final Cox regression models. Place of residence (rural/urban) and use of antihypertensive medications were excluded from the analysis (Fig 2).

### Baseline characteristics

Table 1 illustrated the basic demographic, anthropometric, and laboratory information of the eligible participants. We divided all participants into the training set (n = 12,919) and the testing set (n = 5,535). There were no significant differences in LDL-C, HDL-C, TG, age, BMI, SBP, serum uric acid, creatinine, HR between the two sets (p > 0.05). There were significant differences among participants who living in general deprived areas. As for social, economic and environmental deprived areas, the difference between the training set and the testing set was not statistically significant (p > 0.05).

**Table 1:** Baseline characteristics of the training and testing sets.

Characteristic	Overall, n = 18,454	Training set^1^, n = 12,919	Testing set^1^, n = 5,535	p-value^2^
General deprivation, n (%)^*^				<0.001
Q1	1,821 (9.9)	1,821 (14)	791 (14)	
Q2	3,144 (17)	3,144 (24)	1,389 (25)	
Q3	3,563 (19)	3,563 (28)	1,517 (27)	
Q4	4,391 (24)	4,391 (34)	1,838 (33)	
Social deprivation, n (%)				0.284
Q1	4,483 (24)	3,093 (24)	1,390 (25)	
Q2	4,018 (22)	2,803 (22)	1,215 (22)	
Q3	7,348 (40)	5,181 (40)	2,167 (39)	
Q4	2,605 (14)	1,842 (14)	763 (14)	
Economic deprivation, n (%)				0.608
Q1	3,812 (21)	2,682 (21)	1,130 (20)	
Q2	4,749 (26)	3,335 (26)	1,414 (26)	
Q3	7,651 (41)	5,359 (41)	2,292 (41)	
Q4	2,242 (12)	1,543 (12)	699 (13)	
Environmental deprivation, n (%)				0.701
Q1	1,150 (6.2)	810 (6.3)	340 (6.1)	
Q2	1,462 (7.9)	1,011 (7.8)	451 (8.1)	
Q3	6,981 (38)	4,865 (38)	2,116 (38)	
Q4	8,861 (48)	6,233 (48)	2,628 (47)	
LDL-C (mmol/L), Med (IQR)	3.26 (2.62, 3.95)	3.27 (2.62, 3.96)	3.26 (2.64, 3.93)	0.867
HDL-C (mmol/L), Med (IQR)	1.37 (1.17, 1.62)	1.37 (1.16, 1.63)	1.37 (1.17, 1.62)	0.926
TG (mmol/L), Med (IQR)	1.13 (0.80, 1.65)	1.13 (0.80, 1.67)	1.11 (0.80, 1.63)	0.055
Sex, n (%)				<0.001
Men	5,563 (30)	5,563 (43)	2,373 (43)	
Women	7,356 (40)	7,356 (57)	3,162 (57)	
Age (years), Med (IQR)	44 (34, 54)	44 (34, 54)	44 (34, 53)	0.768
Smoking status, n (%)				<0.001
Current	3,071 (17)	3,071 (24)	3,171 (57)	
Former	2,370 (13)	2,370 (18)	999 (18)	
Never	7,478 (41)	7,478 (58)	1,365 (25)	
BMI (kg/m^2^), Med (IQR)	26.7 (23.4, 30.5)	26.7 (23.4, 30.5)	26.8 (23.5, 30.6)	0.432
SBP (mmHg), Med (IQR)	128 (118, 141)	128 (118, 141)	128 (118, 141)	0.414
Fasting glucose (mmol/L), Med (IQR)	5.10 (4.72, 5.55)	5.10 (4.73, 5.56)	5.10 (4.70, 5.54)	0.045
Serum uric acid (mg/dL), Med (IQR)	5.04 (4.10, 6.10)	5.04 (4.10, 6.10)	5.04 (4.10, 6.11)	0.404
Creatinine (mg/dL), Med (IQR)	69 (62, 76)	69 (62, 77)	69 (62, 76)	0.922
HR (bpm), Med (IQR)	72 (66, 78)	72 (66, 78)	72 (66, 78)	0.303
DM, n (%)				<0.001
No	12,517 (68)	12,517 (97)	5,369 (97)	
Yes	402 (2.2)	402 (3.1)	166 (3.0)	

^1^Quantitative data are expressed as median (Med) with interquartile range (IQR). Categorical data are presented as amounts with percentages.

^2^Comparisons of differences between groups are analyzed by Wilcoxon rank sum test for continuous variables and by Pearson’s Chi-squared test for categorical variables.

*Russian deprivation index measures general deprivation, and its components measure social, economic and environmental deprivation, respectively.

Q1 – the least deprived region; Q4 – the most deprived region.

Q, quantile; SBP, systolic blood pressure; HDL-C, high-density lipoprotein cholesterol; LDL-C, low-density lipoprotein cholesterol; HR, heart rate; BMI, body mass index; TG, triglycerides; DM, diabetes mellitus.

S8 Table showed the baseline characteristics of the two sets by CVD events. The participants with CVD (fatal/nonfatal) had higher age, TG, BMI, SBP, fasting glucose, serum uric acid, creatinine in the training and testing sets (p < 0.05). And there was no statistically significant difference in HDL-C, heart rate in testing set (p > 0.05).

### Multivariate Cox proportional hazard regression analysis

Multivariate analysis showed that smoking status, age, sex, SBP, BMI, LDL-C, HDL-C, and DM were individual-level prognostic factors associated with CVD-free survival in all models (S9 Table). In model 1 general deprivation (p < 0.032, Q2 vs. Q1 (HR 1.19; 95% CI: 1.01–1.39), p < 0.032, Q3 vs. Q1 (HR 1.56; 95% CI: 1.04–2.33)) was associated with CVD-free survival. In model 2 social deprivation (p < 0.003, Q2 vs. Q1 (HR 1.75; 95% CI: 1.21–2.54)) was associated with CVD-free survival. However, in model 3 and 4 economic and environmental deprivation was no statistically significant associated with CVD-free survival.

### Construction of nomograms

**Fig 3:**
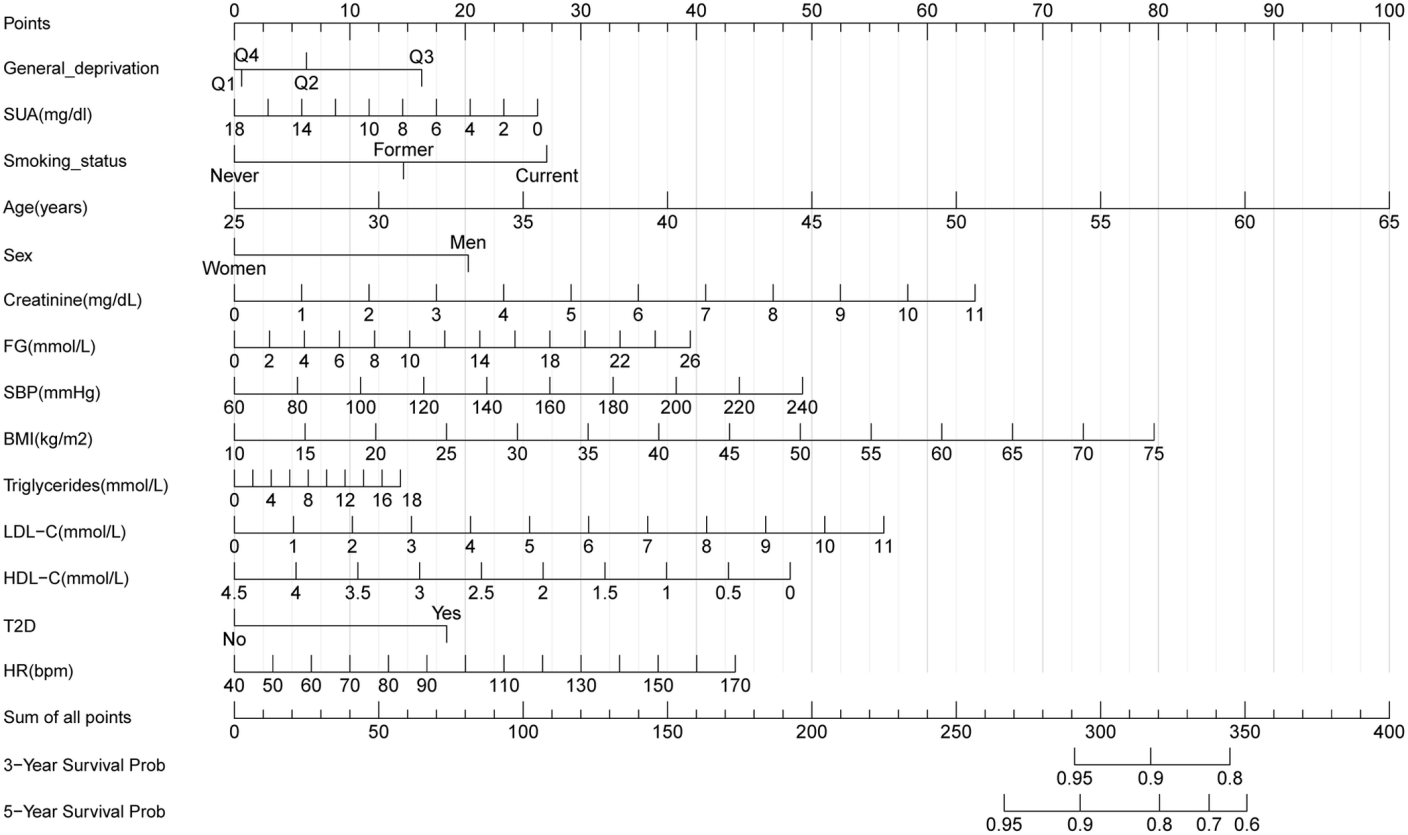
Nomogram A constructed using the independent prognostic factors predicting the three- and five-year CVD-free (fatal/non-fatal) survival. Q, quantile; FG, fasting glucose; SBP, systolic blood pressure; HDL-C, high-density lipoprotein cholesterol; LDL-C, low-density lipoprotein cholesterol; SUA, serum uric acid; T2D, type 2 diabetes mellitus; BMI, body mass index; HR, heart rate.

**Fig 4:**
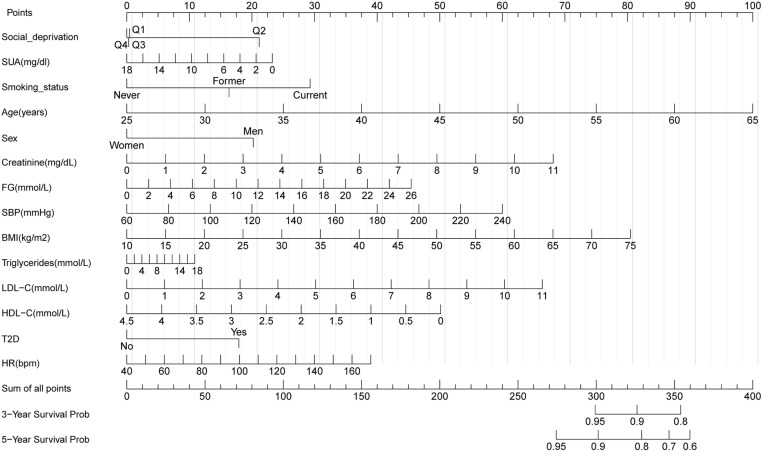
Nomogram B constructed using the independent prognostic factors predicting the three- and five-year CVD-free (fatal/non-fatal) survival. Q, quantile; FG, fasting glucose; SBP, systolic blood pressure; HDL-C, high-density lipoprotein cholesterol; LDL-C, low-density lipoprotein cholesterol; SUA, serum uric acid; T2D, type 2 diabetes mellitus; BMI, body mass index; HR, heart rate.

**Fig 5:**
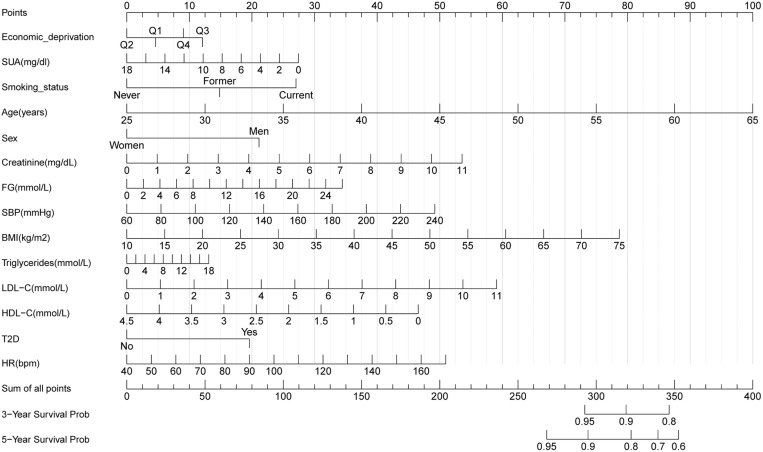
Nomogram C constructed using the independent prognostic factors predicting the three- and five-year CVD-free (fatal/non-fatal) survival. Q, quantile; FG, fasting glucose; SBP, systolic blood pressure; HDL-C, high-density lipoprotein cholesterol; LDL-C, low-density lipoprotein cholesterol; SUA, serum uric acid; T2D, type 2 diabetes mellitus; BMI, body mass index; HR, heart rate.

**Fig 6:**
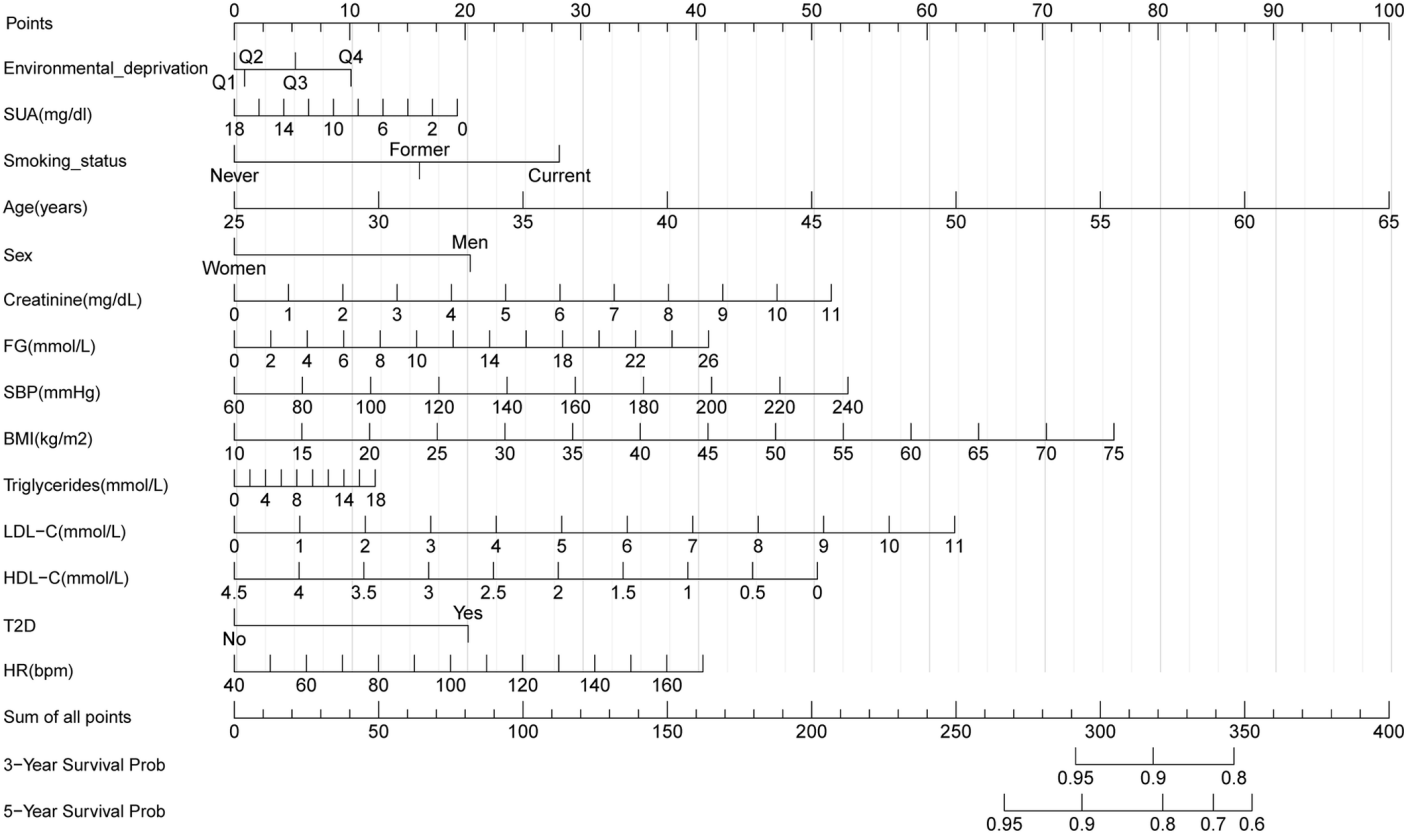
Nomogram D constructed using the independent prognostic factors predicting the three- and five-year CVD-free (fatal/non-fatal) survival. Q, quantile; FG, fasting glucose; SBP, systolic blood pressure; HDL-C, high-density lipoprotein cholesterol; LDL-C, low-density lipoprotein cholesterol; SUA, serum uric acid; T2D, type 2 diabetes mellitus; BMI, body mass index; HR, heart rate.

To predict 3- and 5-year CVD-free survival, four prognostic nomograms were developed from the results of multivariate analysis using the training set (Figs 3–6).

### Validation of the nomograms

**Fig 7:**
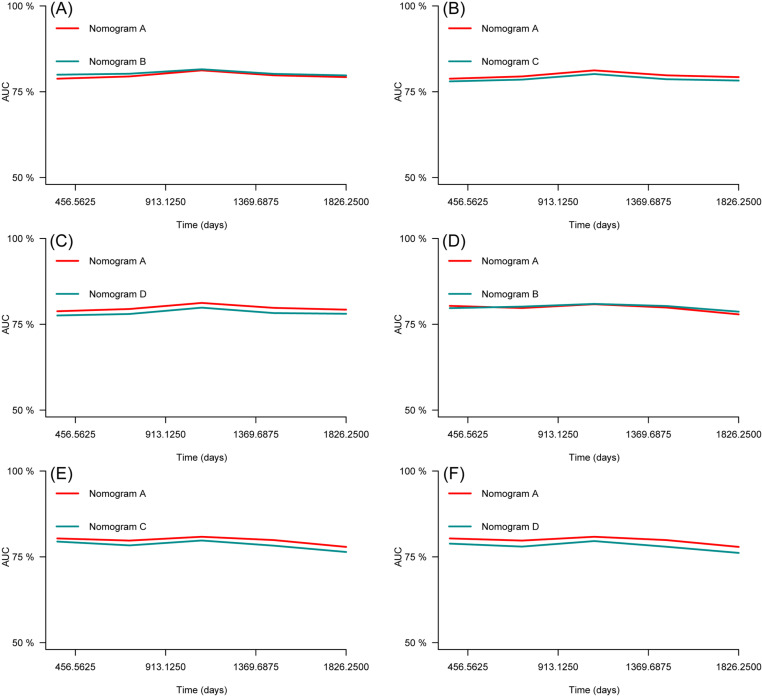
Time-dependent AUC curves of the nomograms for the prediction of CVD-free survival in the training set (A, B, C) and testing set (D, E, F).

**Fig 8:**
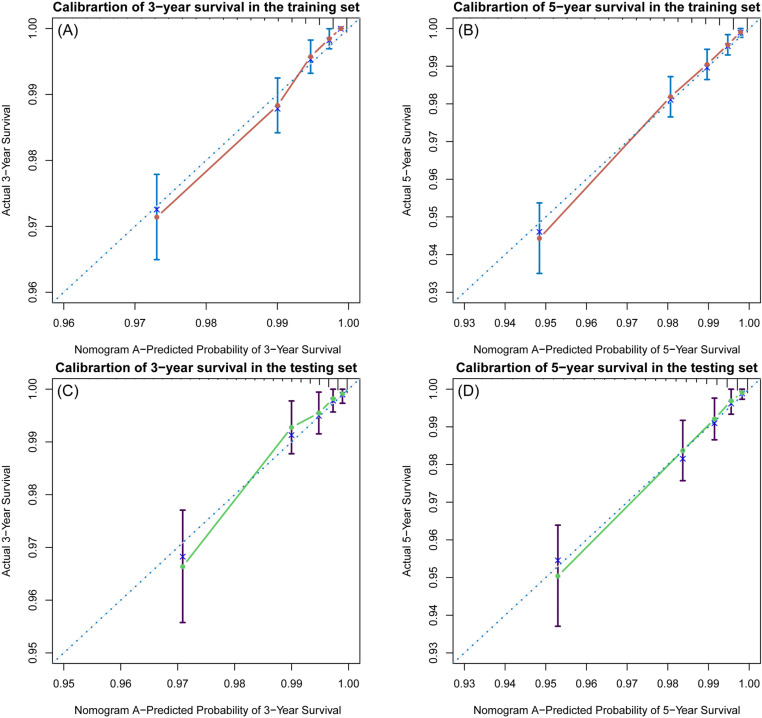
Calibration curves of the nomogram A. **(A)** Three-year and (B) five-year CVD-free (fatal/non-fatal) survival in the training set. **(C)** Three-year and (D) five-year CVD-free survival in the testing set.

**Fig 9:**
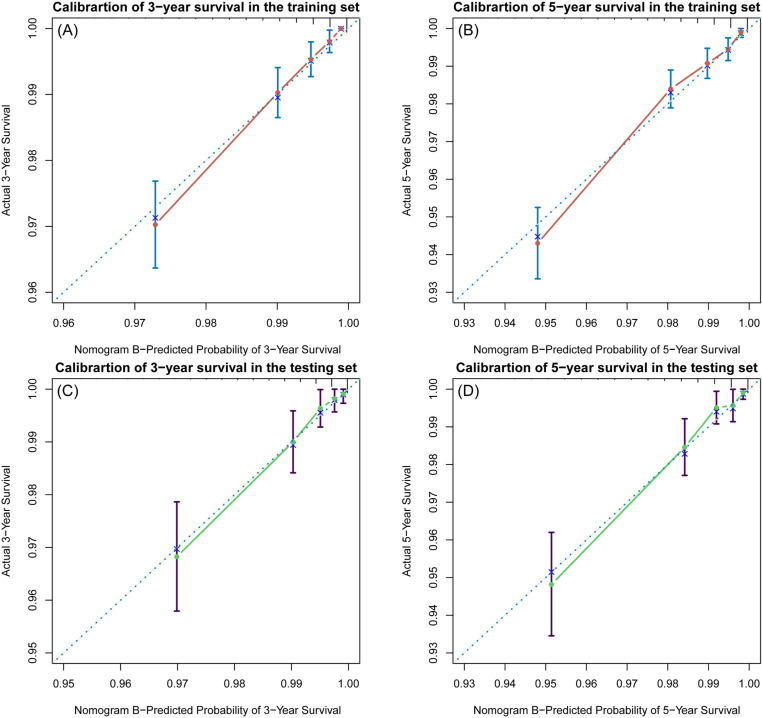
Calibration curves of the nomogram B. **(A)** Three-year and (B) five-year CVD-free (fatal/non-fatal) survival in the training set. **(C)** Three-year and (D) five-year CVD-free survival in the testing set.

**Fig 10:**
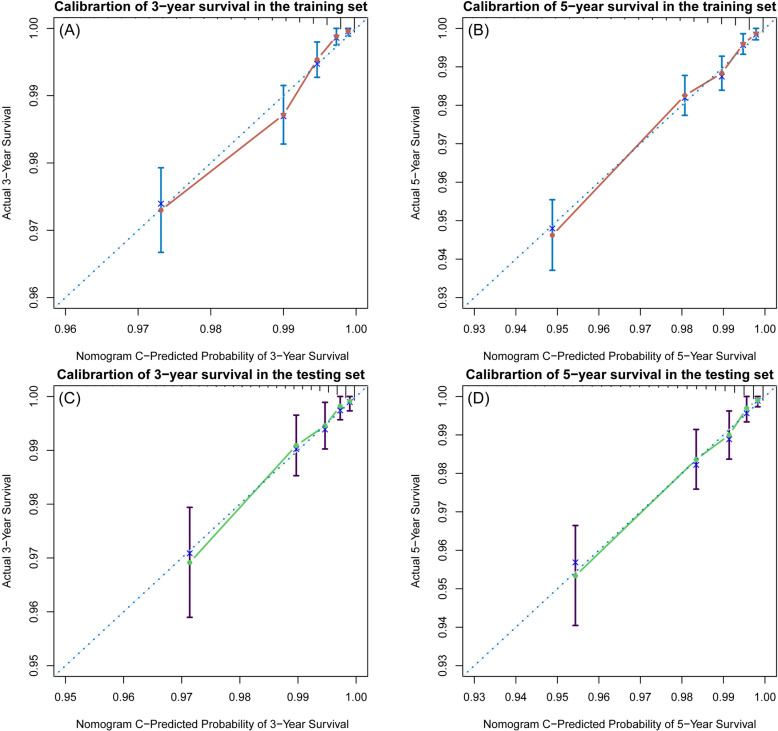
Calibration curves of the nomogram C. **(A)** Three-year and (B) five-year CVD-free (fatal/non-fatal) survival in the training set. **(C)** Three-year and (D) five-year CVD-free survival in the testing set.

**Fig 11:**
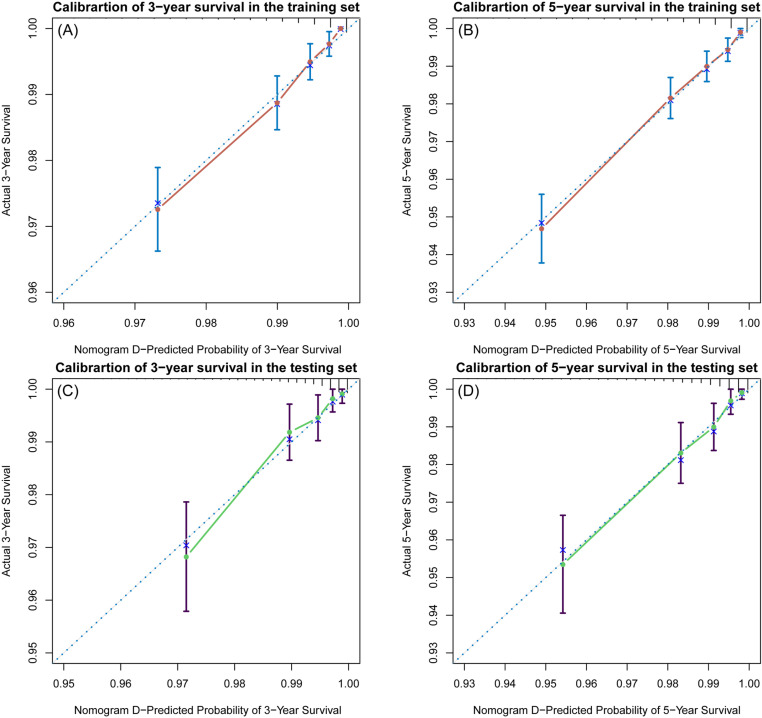
Calibration curves of the nomogram D. **(A)** Three-year and (B) five-year CVD-free (fatal/non-fatal) survival in the training set. **(C)** Three-year and (D) five-year CVD-free survival in the testing set.

**Fig 12:**
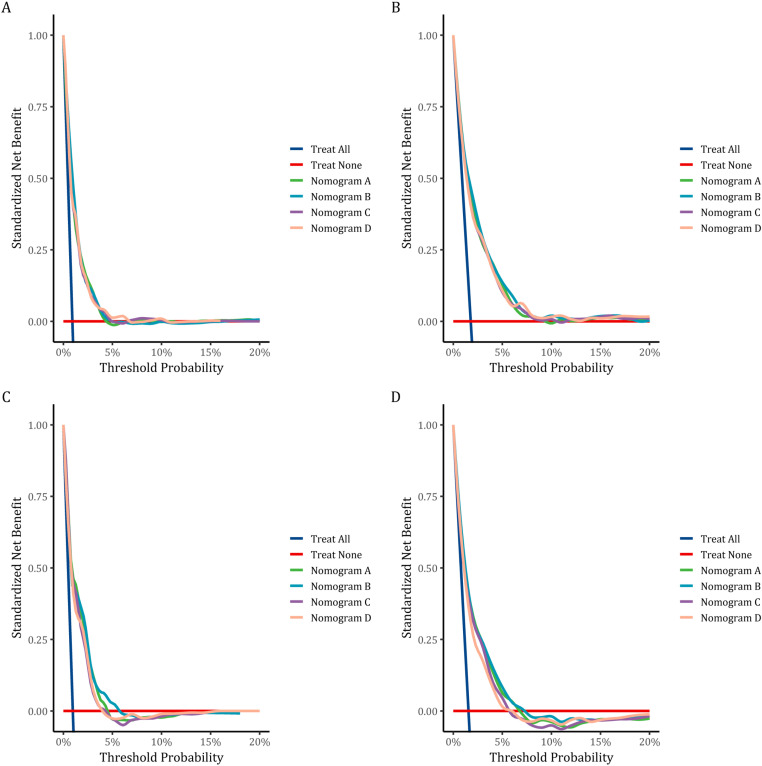
Decision curve analysis of CVD-free survival nomograms. The DCA for three- **(A)**, five-year **(B)**, CVD-free survival prediction in training set and for three - (C) and five-year **(D)** CVD-free survival prediction in the testing set.

The C-indices of the training and testing sets were 0.79 (95% CI: 0.75–0.82) and 0.78 (95% CI: 0.74–0.82), respectively, indicating the great discriminative ability of the nomogram A (Table 2). Other C-index values of the training and testing sets also indicated that nomogram B, C and D had good discriminative ability. The time dependent AUC was > 0.7 for the prediction of CVD-free survival in both the training and testing sets (Fig 7), indicating favorable discrimination by the nomograms. Calibration curves with 1000 bootstrap resamples for 3- and 5-year CVD-free survival were close to the 45-degree diagonal, indicating that the predicted survival probabilities were generally consistent with the observed probabilities (Figs 8–11). Moreover, DCA curves confirmed the validity of the nomograms (Fig 12). In sum, the nomograms for 3- and 5-year CVD-free survival had considerable predictive accuracy and clinical effectiveness.

**Table 2:** Prediction performance of the nomogram for CVD-free survival.

	Training set			Testing set		
	C-index	95% CI		C-index	95% CI	
		Lower	Upper		Lower	Upper
Nomogram A	0.79	0.75	0.82	0.78	0.74	0.82
Nomogram B	0.79	0.76	0.82	0.79	0.75	0.82
Nomogram C	0.78	0.75	0.82	0.77	0.73	0.80
Nomogram D	0.78	0.74	0.81	0.77	0.73	0.81

C-index, Harrell’s concordance-index; CI, confidence interval.

### Comparison of the nomogram A with nomogram B, C and D

**Fig 13:**
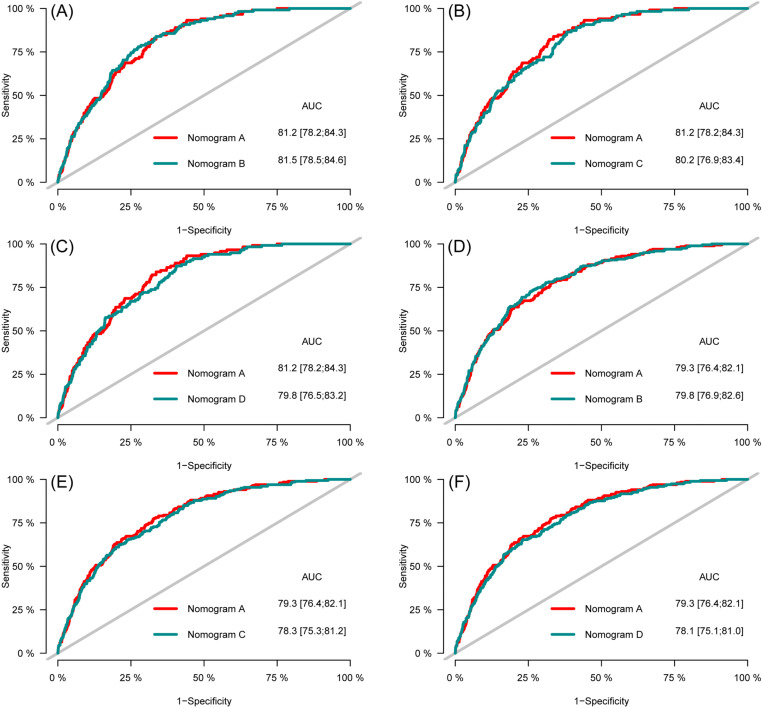
Receiver operating characteristic of the nomograms for three- (A, B, C) and five-year (D, E, F) CVD-free survival prediction in the training set.

**Fig 14:**
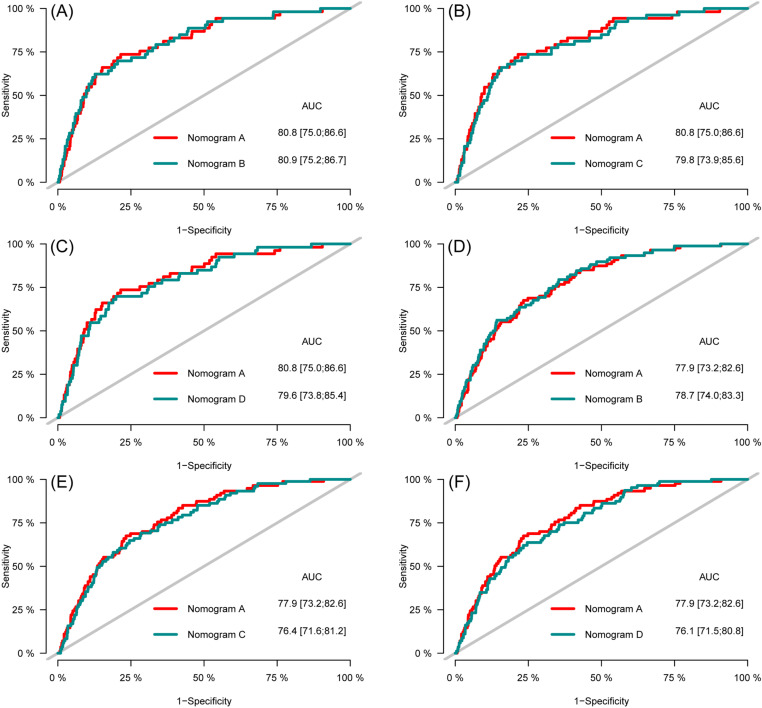
Receiver operating characteristic of the nomograms for three- (A, B, C) and five-year (D, E, F) CVD-free survival prediction in the testing set.

ROC curves were plotted to compare the predictive accuracy of the nomogram A with the nomogram B, C, and D. The comparison of the predictive abilities of the nomogram A with the nomogram B, C and D showed that the AUC values of nomogram A for 3- and 5-year CVD-free survival in the training set (81.2 and 79.3, respectively) were close to the AUC values of nomogram B (81.5 and 79.8, respectively), nomogram C (80.2 and 78.3, respectively), and nomogram D (79.8 and 78.1, respectively) (Fig 13). The results for the testing set also showed that the AUC values of the nomogram A for 3- and 5-year CVD-free survival (80.8 and 77.9, respectively) close to the AUC values for nomogram B (80.9 and 78.7, respectively) nomogram C (79.8 and 76.4, respectively), and nomogram D (79.6, and 76.1, respectively) (Fig 14).

In the accuracy analyses of NRI and IDI, the nomogram A showed similar performance with nomogram B, C and D (Table 3). In the training and testing set, the 3- and 5-year NRI of the nomograms were zero or close to zero. These results together demonstrated that the nomogram A had a same predictive ability with compared to the nomogram B, C and D.

**Table 3:** Comparison of NRI and IDI among nomograms predicting three- and five-year CVD-free survival.

Nomogram	IDI (95% CI)	p-value	NRI (95% CI)
Training set			
3-year CVD-free survival			
Nomogram A	Reference		Reference
Nomogram B	0.000(0.000; 0.001)	0.365	0 (-0.00008; 0.008)
Nomogram C	0.000 (0.000; 0.001)	0.372	0 (-0.0002; 0.02)
Nomogram D	0.000 (0.000; 0.001)	<0.001	0 (-0.0002; 0.01)
5-year CVD-free survival			
Nomogram A	Reference		Reference
Nomogram B	-0.004 (-0.006; -0.001)	0.013	-0.0002 (-0.02; 0.008)
Nomogram C	0.025 (0.020; 0.029)	<0.001	-0.0002 (-0.014; 0.02)
Nomogram D	-0.001(-0.002; 0.000)	0.033	0.004(-0.01; 0.01)
Testing set			
3-year CVD-free survival			
Nomogram A	Reference		Reference
Nomogram B	0.000 (-0.001; 0.000)	0.731	0 (-0.0002; 0.0002)
Nomogram C	0.000 (-0.002; 0.002)	0.738	0 (-0.0004; 0.03)
Nomogram D	0.001 (0.000; 0.001)	0.073	0 (-0.0002; 0.0002)
5-year CVD-free survival			
Nomogram A	Reference		Reference
Nomogram B	-0.004 (-0.006; -0.001)	0.013	0 (-0.001; 0.0007)
Nomogram C	0.027 (0.020; 0.034)	<0.001	0(-0.002; 0.01)
Nomogram D	-0.002(-0.004; 0.000)	0.013	-0.0002(-0.001; 0.001)

CVD, cardiovascular diseases; IDI, integrated discrimination improvement; NRI, net reclassification index; CI, confidence interval.

### Risk group stratification

Beyond the development and validation of the nomograms, all participants in the training set were sorted based on their nomogram scores and classified into three CVD (fatal/non-fatal) risk groups using “rolr” package: low-risk group (total nomogram A points of 0–211, total nomogram B points of 0–216, total nomogram C points of 0–209, total nomogram D points of 0–216), moderate‐risk group (total nomogram A points of 212–244, total nomogram B points of 217–254, total nomogram C points of 210–255, and total nomogram D points of 217–261) and high-risk group (total nomogram A points of 245–400, total nomogram B points of 255–400, total nomogram C points of 256–400, and total nomogram D points of 262–400).

**Fig 15:**
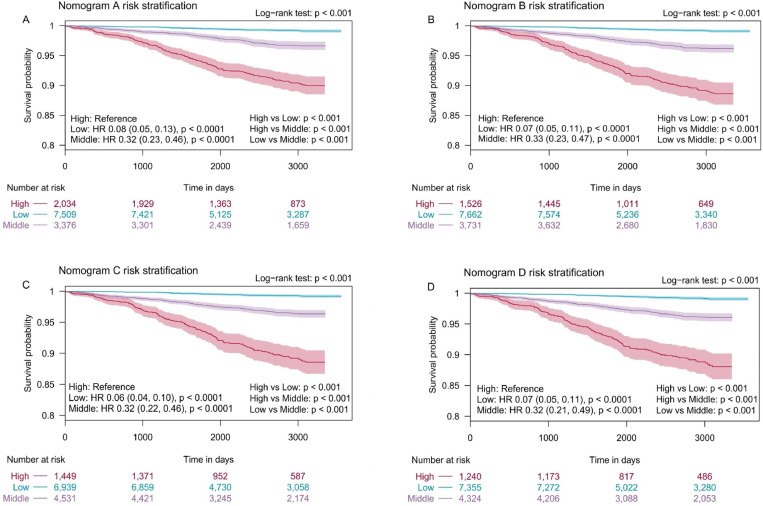
Kaplan–Meier curves of CVD-free survival (fatal/non-fatal) for risk classification based on the nomogram A scores (A), the nomogram B scores (B), the nomogram C scores (C), and the nomogram D scores (D) in the training set. HR, hazard ratio.

The results of the KM survival analysis with the log-rank test showed that there existed different CVD-free survival in three risk groups of participants (p < 0.001) (Fig 15). There were also signiﬁcant differences in pairwise comparisons adjusted by the Bonferroni method among three risk groups.

### Development of a web-based calculator

We utilized “shiny” package to develop a free online calculator based on our nomograms. By using the calculator, the probabilities of 3- and 5-year CVD-free (fatal/non-fatal) survival could be easily obtained. Furthermore, this tool calculates the nomogram total score and determines the risk group. The online calculator can be accessed at https://nomcvdepriv.shinyapps.io/Cardio/

## Discussion

The results of our study demonstrated that area-level indicators were associated with CVD and can be considered as risk factors and included in models for assessing the risk of CVD. As mentioned above, some studies included area-level socio-economic indices as an independent risk factor in their models for assessing CVD risk. In our study has developed the nomogram that includes RDI with both socio-economic (e.g., unemployment rate, low income, etc.), and environmental variables (e.g., fire forest incidence, transport-related emissions from stationary sources: NO_2_, SO_2_, CO, etc.) and the nomograms that include separately the social, economic and environmental components of the index as independent predictors.

Numerous systematic reviews confirmed the association of air pollution indicators with CVD [54–59]. Basic pathophysiological mechanisms, which are activated by exposure to air pollutants and promotes adverse cardiovascular effects are systemic inflammation, oxidative stress, thrombosis, coagulation, vascular dysfunction and atherosclerosis [60–64].

Important is the fact that nomograms created in our study were validated in the Russian population. As mentioned above, we know only two CVD risk assessment scores that have been validated in the Russian population – SCORE and SCORE-2. There are not area-level independent predictors in both scores. Consequently, our study is the first to create nomograms that included individual-level and area-level independent predictors and validated in Russian population. In addition, our nomograms, unlike these CVD risk assessment scores where total cholesterol is used as an independent predictor, included lipid spectrum indicators, such as TG, HDL-C and LDL-C. Our nomograms demonstrated that low levels of HDL-C and high levels of TG and LDL-C contribute significantly to the risk of CVD. This result is similar to the findings of Bartlett et al. [65], who examined the association of low levels of HDL-C with a risk of CVD against the background of increased levels of TG and LDL-C and found that CVD risk was higher when low HDL-C was accompanied by LDL-C ≥ 100 mg/dL (OR 1.3, 95% CI: 1.0–1.6), TG ≥ 100 mg/dL (OR 1.3, 95% CI: 1.1–1.5) or both (OR 1.6, 95% CI: 1.2–2.2). Moreover, the study shown that high levels of TG increase the risk of developing CVD, regardless of the level of HDL-C and LDL-C. Another study [66] also found that increasing TG levels increased the risk of CVD, regardless of HDL-C levels, and in men there was an increase in the risk of CVD with increasing TG levels to 100 mg/dL, and in women to 200 mg/dL. It follows that hypertriglyceridemia is a powerful risk factor for adverse cardiovascular events. This fact may be taken into account when prescribing lipid-lowering medications [67–69]. Therefore, our nomograms can not only assess the risk of CVD, but also contribute to the development of more effective recommendations aimed at preventing CVD, in contrast to the scores that include only total cholesterol.

Our nomograms demonstrated that with increasing BMI also increases the risk of CVD. Kim et al. [70] conducted an umbrella review that included 12 systematic reviews with 53 meta-analyses, which covering more than 501 cohorts and 30 million participants, also confirmed that an increase in BMI was associated with a higher risk of developing all specific CVD.

Our nomograms include blood glucose levels, which allows us to assess the impact of prediabetes on the occurrence of CVD. Moreover, the results of our study demonstrated an increase in the risk of CVD with increasing glucose levels. Our findings is supported by a prospective study [71] conducted in northeast China among 15,557 participants without diabetes aged over 40 years, which found that multivariate-adjusted hazard ratios for CVD mortality in groups with prediabetes and hypertension were 2.28 (95% CI: 1.50–3.47) for those diagnosed by fasting plasma glucose (FPG) < 5.6 mmol/L, 2.18 (95% CI: 1.53–3.10) for those diagnosed by FPG 5.6–6.0 mmol/L and 2.35 (95% CI: 1.65–3.35) for those diagnosed by FPG 6.1–6.9 mmol/L compared with the reference group.

Another study [72] also found that prediabetes (126 mg/dL > FPG ≥ 100 mg/dL) was associated with a 1.95-fold (95% CI: 1.43–2.52, p < 0.0001) increased risk for hypertension. Huang et al. [73] conducted a systematic review with meta-analysis and confirmed that prediabetes was associated with an increased risk of cardiovascular disease. Another systematic review with meta-analysis [74] included total of 129 studies involving 10,069,955 individuals for analysis revealed that in the general population, prediabetes was associated with an increased risk of composite CVD (relative risk (RR) 1.15, 95% CI: 1.11–1.18), CHD (RR 1.16, 95% CI: 1.11–1.21), and stroke (RR 1.14, 95% CI: 1.08–1.20). In patients with ASCVD, prediabetes was associated with an increased risk of composite CVD (RR 1.37, 95% CI: 1.23–1.53), and CHD (RR 1.15, 95% CI: 1.02–1.29). Cao et al. [75] found that progression from prediabetes to diabetes within a 3-year period was associated with higher risks of CVD-related death (HR 1.61, 95% CI: 1.12–2.33) compared with persistent prediabetes. Kim et al. [76] found that individuals with normal fasting glucose (< 5.5 mmol/L) at baseline, who were subsequently newly diagnosed with diabetes (fasting glucose: ≥ 7.0 mmol/L) and prediabetes (fasting glucose: 5.5–6.9 mmol/L), had increased CVD incidence (HR 1.13, (95% CI: 1.05–1.23) and HR 1.04 (95% CI: 1.01–1.07), respectively).

In our nomograms, lowering uric acid levels increases the risk of CVD. Although hyperurecemia is believed to be a risk factor for CVD, the role of uric acid as an independent predictor remains controversial. These contradictions may be associated with pathophysiological processes involving uric acid, which lead to the occurrence and development of CVD [77]. On the one hand, uric acid acts as an antioxidant; on the other hand, uric acid represents a pro-oxidant agent [78]. Acting as pro-oxidant agent a high level of uric acid triggers inflammation and oxidation process (oxidative stress), resulting in endothelial dysfunction, which precedes the development of atherosclerosis [79]. However, uric acid as an antioxidant has protective properties in relation to the vascular endothelium and low levels of uric acid can also activate mechanisms leading to endothelial dysfunction. These assumptions are confirmed by numerous studies. For instance, a study conducted in Japan [80] found U-shaped association between serum uric acid level and risk of CVD mortality. The multivariable adjusted HRs for CVD mortality comparing uric acid < 2.5 mg/dL with uric acid of 3.5–4.4 mg/dL were 3.96 (95% CI: 1.37–11.47) in women. At the same time, in women with uric acid concentrations of ≥ 8.5 mg/dL also had a greater risk for mortality from CVD (HR 11.44, 95% CI: 2.74–47.68), in comparison to women with uric acid concentrations of 3.5–4.4 mg/dL. Another study [81] included 127,771 adults aged 65 years or older also established that SUA ≥ 8 mg/dL (HR 1.13, 95% CI: 1.06–1.21) or < 4 mg/dL (HR 1.16, 95% CI: 1.07–1.25) compared with the reference SUA strata of 4 to < 5 mg/dL independently predicted higher CVD‐related mortality. Some studies also revealed U-shaped or non-linear association between serum uric acid level and risk of CVD mortality [82–84], hypertensive heart failure [85], ischemic stroke [86], and other cardiovascular events [87,88] Also, a number of studies found that low uric acid level predicted poor functional outcomes in acute stroke [89,90]. In any case, the influence of uric acid levels on the occurrence and development of CVD requires further investigation.

The results of our study shown that high creatinine levels increase the risk of CVD. Schytz et al. [91] demonstrated that increasing creatinine from 0 to 30% increased the risk of cardiovascular event among men aged 50–79 years. Chen et al. [92] shown that serum creatinine levels were positively associated with 10-year cardiovascular risk (β = 0.432, p < 0.001) in total participants.

Our results, as well as previous numerous studies [93–96], confirmed the harmful effects of tobacco on the cardiovascular system. The mechanisms by which smoking results in cardiovascular events have been well studied [97]. It has now been established that there is a genetic predisposition to smoking [98–100]. It can be assumed that this fact is one of the reasons for the ineffectiveness of many strategies aimed at preventing and reducing tobacco use [101–103].

### Strengths and limitations

This study has some limitations. First, information about medications, including diuretics, lipid-lowering and urate-lowering agents, was not included in this study. Secondly, there is no data available to estimate the 10-year probability CVD-free survival. Nevertheless, our study has several strengths. For the first time, nomograms have been created that include area-level predictors (socio-economic and environmental) and lipid spectrum indicators (TG, HDL-C and LDL-C) and assess the probability of fatal and non-fatal cardiovascular events among the Russian population. Finally, our nomograms were validated using data of population-based nationwide study – ESSE-RF that included the largest representative sample of the general population of Russia.

## Conclusions

It is widely understood that preventing and detecting the disease at an early stage of its development are the most effective measures to fight against it. Our nomograms can provide an early prediction for the risk of new-onset cardiovascular disorders as well as timely intervention measures to prevent or delay the occurrence of CVD, and ultimately reduce the adverse cardiovascular prognosis among Russian adults.

## Supporting information

S1 Table TRIPOD (Transparent Reporting of a multivariable prediction model for Individual Prognosis or Diagnosis) checklist. (DOCX)

S2 Table Components of Russian deprivation index. (DOCX)

S3 Table Definitions of deprivation indicators. (DOCX)

S4 Table The federal subjects of Russia stratified by level of general deprivation. (DOCX)

S5 Table The federal subjects of Russia stratified by level of social deprivation. (DOCX)

S6 Table The federal subjects of Russia stratified by level of economic deprivation. (DOCX)

S7 Table The federal subjects of Russia stratified by level of environmental deprivation. (DOCX)

S8 Table Baseline characteristics for the training and testing sets by CVD event (fatal/non-fatal). (DOCX)

S9 Table Multivariate analysis of CVD-free survival in the training set. (DOCX)
