# Supplementary material for: Development and validation of nomograms including individual- and area-level variables to predict risk of fatal and non-fatal cardiovascular diseases among Russian population
Source: PLoS One. 2025 Jun 2;20(5):e0324736. doi: 10.1371/journal.pone.0324736 (PMC12129350; doi:10.1371/journal.pone.0324736)
Supplement: S2 Table — (DOCX) [file pone.0324736.s002.docx]

**S Table 1. Components of Russian deprivation index.**

| Social deprivation | Economic deprivation | Environmental deprivation |
| --- | --- | --- |
| Living in crowded households | Stove heating | Dead forest |
| Families with children under age 5 years old | No hot water supply | Fire forest incidence |
| Families with 3 and more children (aged 0‒18) | No sewerage system | Environmental crime |
| Unemployment rate | Not central sewerage system | Transport-related emissions |
| Households with phone | Low income | Emissions from stationary sources: NO_2_, SO_2_, CO |
