# Supplementary material for: Development and validation of nomograms including individual- and area-level variables to predict risk of fatal and non-fatal cardiovascular diseases among Russian population
Source: PLoS One. 2025 Jun 2;20(5):e0324736. doi: 10.1371/journal.pone.0324736 (PMC12129350; doi:10.1371/journal.pone.0324736)
Supplement: S3 Table — (DOCX) [file pone.0324736.s003.docx]

**S Table 2. Definitions of deprivation indicators.**

| Domain | Variable | Description | Data source |
| --- | --- | --- | --- |
| Family structure/Demographics | Children +3 | Percentage of families with 3 and more children (aged 0‒18) | Census 2010 |
|  | Children under 5 years old | Children aged 0‒4 as a percentage of total population | Census 2010 |
| Housing | Stove heating | Percentage of households with stove heating | Census 2010 |
|  | No hot water supply | Percentage of households without heat water supply | Census 2010 |
|  | No central sewerage system | Percentage of households with toilets emptying into a cesspit | Census 2010 |
|  | No sewerage system | Percentage of households without sewage system | Census 2010 |
|  | Overcrowded | Percentage of households (individual (single-family) houses, individual and communal apartments) with > 5 persons | Census 2010 |
| Communication | Phone | Percentage of households with telephone | Census 2010 |
| Income and Wealth | Low income | Percentage of people below a low income threshold in the total population | Regions of Russia. Social and Economic Indicators – 2011 |
|  | Unemployment rate | Population 15 or older unemployed | Labour and Employment in Russia – 2011 |
| Air quality | NO_2_ | Nitrogen dioxide (thousand tons) from stationary sources | Environment Protection in Russia – 2012 |
|  | SO_2_ | Sulphur dioxide (thousand tons) from stationary sources | Environment Protection in Russia – 2012 |
|  | CO | Carbon monoxide (thousand tons) from stationary sources | Environment Protection in Russia – 2012 |
|  | Transport-related emissions | Air emissions from vehicle (thousand tons) | Environment Protection in Russia – 2012 |
| Natural disaster | Fire forest incidence | The number of fire forest incidence (unit) | Environment Protection in Russia – 2012 |
| Green space | Area of dead forest | The area of dead forest (hectares) | Environment Protection in Russia – 2012 |
| Crimes | Environmental crimes | The number of recorded environmental crimes | Environment Protection in Russia – 2012 |
