# Supplementary material for: Development and validation of nomograms including individual- and area-level variables to predict risk of fatal and non-fatal cardiovascular diseases among Russian population
Source: PLoS One. 2025 Jun 2;20(5):e0324736. doi: 10.1371/journal.pone.0324736 (PMC12129350; doi:10.1371/journal.pone.0324736)
Supplement: S4 Table — (DOCX) [file pone.0324736.s004.docx]

**S4 Table. The Russian federal subjects stratified by level of general deprivation.**

| **Level of general deprivation** | **The Russian federal subjects** |
| --- | --- |
| Q1 – the least deprived areas | Belgorod Region, Vladimir Region, Ivanovo Region, Kabardino-Balkarian Republic, Kaliningrad Region, Kaluga Region, Kamchatka Territory, Leningrad Region, Magadan Region, Moscow, Murmansk Region, Oryol Region, Republic of Adygea, Republic of Mordovia, St. Petersburg, Stavropol Territory, Tula Region, Chukotka Autonomous Area, Yaroslavl Region, Republic of North Ossetia - Alania |
| Q2 | Bryansk Region, Voronezh Region, Karachayevo-Circassian Republic, Krasnodar Territory, Kursk Region, Lipetsk Region, Moscow Region, Nenets Autonomous Area, Novgorod Region, Penza Region, Pskov Region, Republic of Kalmykia, Mari El Republic, Ryazan Region, Samara Region, Sakhalin Region, Smolensk Region, Tambov Region, Ulyanovsk Region, Chuvash Republic, Yamal-Nenets Autonomous Area |
| Q3 | Jewish Autonomous Region, Astrakhan Region, Volgograd Region, Vologda Region, Kirov Region, Kostroma Region, Nizhny Novgorod Region, Novosibirsk Region, Perm Territory, Primorye Territory, Republic of Karelia, Komi Republic, Republic of Tatarstan, Republic of Khakassia, Rostov Region, Saratov Region, Tver Region, Tomsk Region, Udmurtian Republic, Khabarovsk Territory, Khanty-Mansi Autonomous Area - Yugra |
| Q4 – the most deprived areas | Altai Territory, Amur Region, Arkhangelsk Region, Trans-Baikal Territory, Irkutsk Region, Kemerovo Region,  Krasnoyarsk Territory, Kurgan Region, Omsk Region, Orenburg Region, Altai Republic, Republic of Bashkortostan,  Republic of Buryatia, Republic of Daghestan,  Republic of Ingushetia, Republic of Sakha (Yakutia), Tuva Republic, Sverdlovsk Region, Tyumen Region, Chelyabinsk Region, Chechen Republic |
