# Supplementary material for: Development and validation of nomograms including individual- and area-level variables to predict risk of fatal and non-fatal cardiovascular diseases among Russian population
Source: PLoS One. 2025 Jun 2;20(5):e0324736. doi: 10.1371/journal.pone.0324736 (PMC12129350; doi:10.1371/journal.pone.0324736)
Supplement: S5 Table — (DOCX) [file pone.0324736.s005.docx]

**S5 Table. The Russian federal subjects stratified by level of social deprivation.**

| **Level of social deprivation** | **The Russian federal subjects** |
| --- | --- |
| Q1 – the least deprived areas | Vladimir Region, Voronezh Region, Ivanovo Region, Kaluga Region, Kamchatka Territory, Kostroma Region, Magadan Region, Moscow, Nizhny Novgorod Region, Novgorod Region, Oryol Region, Penza Region, Pskov Region, Mari El Republic, Republic of Mordovia, Ryazan Region, St. Petersburg, Smolensk Region, Ulyanovsk Region, Yaroslavl Region |
| Q2 | Jewish Autonomous Region, Altai Territory, Bryansk Region,  Kaliningrad Region, Kirov Region, Kurgan Region, Kursk Region, Leningrad Region, Moscow Region, Murmansk Region, Novosibirsk Region, Primorye Territory, Republic of Kalmykia, Republic of Karelia, Samara Region, Sakhalin Region, Tambov Region, Tver Region, Tula Region, Khabarovsk Territory, Chuvash Republic |
| Q3 | Amur Region, Arkhangelsk Region, Belgorod Region, Volgograd Region, Vologda Region, Krasnodar Territory, Krasnoyarsk Territory, Lipetsk Region, Omsk Region, Orenburg Region, Perm Territory, Republic of Adygea, Komi Republic, Republic of Tatarstan, Republic of Khakassia, Rostov Region, Saratov Region, Stavropol Territory, Tomsk Region, Udmurtian Republic, Chukotka Autonomous Area |
| Q4 – the most deprived areas | Astrakhan Region, Trans-Baikal Territory, Irkutsk Region, Kabardino-Balkarian Republic, Karachayevo-Circassian Republic, Kemerovo Region, Nenets Autonomous Area, Altai Republic, Republic of Bashkortostan, Republic of Buryatia, Republic of Daghestan, Republic of Ingushetia, Republic of Sakha (Yakutia), Tuva Republic, Sverdlovsk Region, Tyumen Region, Khanty-Mansi Autonomous Area - Yugra, Chelyabinsk Region, Chechen Republic, Yamal-Nenets Autonomous Area, Republic of North Ossetia - Alania |
