# Supplementary material for: Development and validation of nomograms including individual- and area-level variables to predict risk of fatal and non-fatal cardiovascular diseases among Russian population
Source: PLoS One. 2025 Jun 2;20(5):e0324736. doi: 10.1371/journal.pone.0324736 (PMC12129350; doi:10.1371/journal.pone.0324736)
Supplement: S6 Table — (DOCX) [file pone.0324736.s006.docx]

**S6 Table. The Russian federal subjects stratified by level of economic deprivation.**

| **Level of economic deprivation** | **The Russian federal subjects** |
| --- | --- |
| Q1 – the least deprived areas | Belgorod Region, Kabardino-Balkarian Republic, Kaliningrad Region, Krasnodar Territory, Leningrad Region,  Magadan Region, Moscow, Moscow Region, Murmansk Region, Republic of Tatarstan, Samara Region, St. Petersburg, Sakhalin Region, Sverdlovsk Region, Stavropol Territory, Tula Region, Khanty-Mansi Autonomous Area - Yugra, Chukotka Autonomous Area, Yamal-Nenets Autonomous Area, Republic of North Ossetia - Alania |
| Q2 | Astrakhan Region, Volgograd Region, Kaluga Region, Kamchatka Territory, Karachayevo-Circassian Republic, Kemerovo Region, Lipetsk Region, Nenets Autonomous Area, Nizhny Novgorod Region, Orenburg Region, Perm Territory, Republic of Adygea, Republic of Ingushetia, Komi Republic, Rostov Region, Ryazan Region, Tyumen Region, Khabarovsk Territory, Chelyabinsk Region, Chechen Republic, Yaroslavl Region |
| Q3 | Arkhangelsk Region, Bryansk Region, Vladimir Region,  Vologda Region, Ivanovo Region, Krasnoyarsk Territory,  Kursk Region, Novosibirsk Region, Oryol Region, Penza Region, Primorye Territory, Republic of Bashkortostan, Republic of Daghestan, Republic of Karelia, Saratov Region, Smolensk Region, Tambov Region, Tver Region, Tomsk Region, Udmurtian Republic, Ulyanovsk Region |
| Q4 – the most deprived areas | Jewish Autonomous Region, Altai Territory, Amur Region,  Voronezh Region, Trans-Baikal Territory, Irkutsk Region, Kirov Region, Kostroma Region, Kurgan Region, Novgorod Region, Omsk Region, Pskov Region, Altai Republic, Republic of Buryatia, Republic of Kalmykia, Mari El Republic, Republic of Mordovia, Republic of Sakha (Yakutia), Tuva Republic, Republic of Khakassia, Chuvash Republic |
