# Supplementary material for: Development and validation of nomograms including individual- and area-level variables to predict risk of fatal and non-fatal cardiovascular diseases among Russian population
Source: PLoS One. 2025 Jun 2;20(5):e0324736. doi: 10.1371/journal.pone.0324736 (PMC12129350; doi:10.1371/journal.pone.0324736)
Supplement: S7 Table — (DOCX) [file pone.0324736.s007.docx]

**S7 Table. The Russian federal subjects stratified by level of environmental deprivation.**

| **Level of environmental deprivation** | **The Russian federal subjects** |
| --- | --- |
| Q1 – the least deprived areas | Jewish Autonomous Region, Kabardino-Balkarian Republic, Kaliningrad Region, Kaluga Region, Kamchatka Territory, Karachayevo-Circassian Republic, Kursk Region, Magadan Region, Nenets Autonomous Area, Oryol Region, Republic of Adygea, Altai Republic, Republic of Ingushetia, Republic of Kalmykia, Republic of Mordovia, Tuva Republic, St. Petersburg, Chuvash Republic, Chukotka Autonomous Area, Republic of North Ossetia - Alania |
| Q2 | Belgorod Region, Bryansk Region, Vladimir Region,  Ivanovo Region, Kostroma Region, Moscow, Murmansk Region, Novgorod Region, Penza Region, Pskov Region, Republic of Daghestan, Mari El Republic, Republic of Khakassia, Saratov Region, Sakhalin Region, Smolensk Region, Stavropol Territory, Tambov Region, Tula Region, Ulyanovsk Region, Yaroslavl Region |
| Q3 | Amur Region, Astrakhan Region, Volgograd Region,  Voronezh Region, Kirov Region, Krasnodar Territory,  Kurgan Region, Leningrad Region, Lipetsk Region, Republic of Karelia, Republic of Sakha (Yakutia),  Republic of Tatarstan, Rostov Region, Ryazan Region,  Samara Region, Tver Region, Tomsk Region, Udmurtian Republic, Khabarovsk Territory, Chechen Republic, Yamal-Nenets Autonomous Area |
| Q4 – the most deprived areas | Altai Territory, Arkhangelsk Region, Vologda Region,  Trans-Baikal Territory, Irkutsk Region, Kemerovo Region, Krasnoyarsk Territory, Moscow Region, Nizhny Novgorod Region, Novosibirsk Region, Omsk Region, Orenburg Region, Perm Territory, Primorye Territory, Republic of Bashkortostan, Republic of Buryatia, Komi Republic, Sverdlovsk Region, Tyumen Region, Khanty-Mansi Autonomous Area - Yugra, Chelyabinsk Region |
