# Supplementary material for: Development and validation of nomograms including individual- and area-level variables to predict risk of fatal and non-fatal cardiovascular diseases among Russian population
Source: PLoS One. 2025 Jun 2;20(5):e0324736. doi: 10.1371/journal.pone.0324736 (PMC12129350; doi:10.1371/journal.pone.0324736)
Supplement: S8 Table — (DOCX) [file pone.0324736.s008.docx]

**S8 Table. Baseline characteristics for the training and testing sets by CVD event (fatal/non-fatal).**

| Characteristic | Training set^1^ | | | | Testing set^1^ | | | |
| --- | --- | --- | --- | --- | --- | --- | --- | --- |
|  | Overall, n=12,919 | non-CVD event, n=12,607 | CVD event, n=312 | p-value^2^ | Overall, n=5,535 | non-CVD event, n=5,402 | CVD event, n=133 | p-value^2^ |
| General deprivation* |  |  |  | 0.016 |  |  |  | 0.045 |
| Q1 | 1,821 (14%) | 1,778 (14%) | 43 (14%) |  | 791 (14%) | 775 (14%) | 16 (12%) |  |
| Q2 | 3,144 (24%) | 3,082 (24%) | 62 (20%) |  | 1,389 (25%) | 1,356 (25%) | 33 (25%) |  |
| Q3 | 3,563 (28%) | 3,453 (27%) | 110 (35%) |  | 1,517 (27%) | 1,467 (27%) | 50 (38%) |  |
| Q4 | 4,391 (34%) | 4,294 (34%) | 97 (31%) |  | 1,838 (33%) | 1,804 (33%) | 34 (26%) |  |
| Social deprivation |  |  |  | 0.042 |  |  |  | 0.003 |
| Q1 | 3,093 (24%) | 3,021 (24%) | 72 (23%) |  | 1,390 (25%) | 1,361 (25%) | 29 (22%) |  |
| Q2 | 2,803 (22%) | 2,721 (22%) | 82 (26%) |  | 1,215 (22%) | 1,171 (22%) | 44 (33%) |  |
| Q3 | 5,181 (40%) | 5,076 (40%) | 105 (34%) |  | 2,167 (39%) | 2,130 (39%) | 37 (28%) |  |
| Q4 | 1,842 (14%) | 1,789 (14%) | 53 (17%) |  | 763 (14%) | 740 (14%) | 23 (17%) |  |
| Economic deprivation |  |  |  | 0.101 |  |  |  | 0.333 |
| Q1 | 2,682 (21%) | 2,629 (21%) | 53 (17%) |  | 1,130 (20%) | 1,100 (20%) | 30 (23%) |  |
| Q2 | 3,335 (26%) | 3,261 (26%) | 74 (24%) |  | 1,414 (26%) | 1,380 (26%) | 34 (26%) |  |
| Q3 | 5,359 (41%) | 5,209 (41%) | 150 (48%) |  | 2,292 (41%) | 2,233 (41%) | 59 (44%) |  |
| Q4 | 1,543 (12%) | 1,508 (12%) | 35 (11%) |  | 699 (13%) | 689 (13%) | 10 (7.5%) |  |
| Environmental deprivation |  |  |  | 0.03 |  |  |  | 0.341 |
| Q1 | 810 (6.3%) | 790 (6.3%) | 20 (6.4%) |  | 340 (6.1%) | 330 (6.1%) | 10 (7.5%) |  |
| Q2 | 1,011 (7.8%) | 988 (7.8%) | 23 (7.4%) |  | 451 (8.1%) | 445 (8.2%) | 6 (4.5%) |  |
| Q3 | 4,865 (38%) | 4,771 (38%) | 94 (30%) |  | 2,116 (38%) | 2,068 (38%) | 48 (36%) |  |
| Q4 | 6,233 (48%) | 6,058 (48%) | 175 (56%) |  | 2,628 (47%) | 2,559 (47%) | 69 (52%) |  |
| LDL-C (mmol/L) | 3.27 (2.62, 3.96) | 3.25 (2.61, 3.95) | 3.75 (3.08, 4.51) | <0.001 | 3.26 (2.64, 3.93) | 3.24 (2.64, 3.92) | 3.59 (3.05, 4.25) | <0.001 |
| HDL-C (mmol/L) | 1.37 (1.16, 1.63) | 1.37 (1.17, 1.63) | 1.29 (1.11, 1.52) | <0.001 | 1.37 (1.17, 1.62) | 1.37 (1.17, 1.62) | 1.33 (1.13, 1.58) | 0.22 |
| TG (mmol/L) | 1.13 (0.80, 1.67) | 1.12 (0.80, 1.66) | 1.41 (1.01, 2.18) | <0.001 | 1.11 (0.80, 1.63) | 1.11 (0.79, 1.62) | 1.31 (0.99, 1.89) | <0.001 |
| Sex |  |  |  | <0.001 |  |  |  | <0.001 |
| Men | 5,563 (43%) | 5,379 (43%) | 184 (59%) |  | 2,373 (43%) | 2,293 (42%) | 80 (60%) |  |
| Women | 7,356 (57%) | 7,228 (57%) | 128 (41%) |  | 3,162 (57%) | 3,109 (58%) | 53 (40%) |  |
| Age (years) | 44 (34, 54) | 44 (34, 54) | 54 (49, 59) | <0.001 | 44 (34, 53) | 44 (34, 53) | 54 (50, 59) | <0.001 |
| Smoking status |  |  |  | <0.001 |  |  |  | 0.011 |
| Current | 3,071 (24%) | 2,964 (24%) | 107 (34%) |  | 3,171 (57%) | 3,109 (58%) | 62 (47%) |  |
| Former | 2,370 (18%) | 2,298 (18%) | 72 (23%) |  | 999 (18%) | 975 (18%) | 24 (18%) |  |
| Never | 7,478 (58%) | 7,345 (58%) | 133 (43%) |  | 1,365 (25%) | 1,318 (24%) | 47 (35%) |  |
| BMI (kg/m^2^) | 26.7 (23.4, 30.5) | 26.7 (23.3, 30.5) | 29.4 (25.6, 33.4) | <0.001 | 26.8 (23.5, 30.6) | 26.8 (23.4, 30.5) | 28.5 (25.9, 32.9) | <0.001 |
| SBP (mmHg) | 128 (118, 141) | 128 (118, 140) | 141 (126, 155) | <0.001 | 128 (118, 141) | 128 (118, 141) | 141 (128, 155) | <0.001 |
| Fasting glucose (mmol/L) | 5.10 (4.73, 5.56) | 5.10 (4.73, 5.54) | 5.33 (4.95, 6.15) | <0.001 | 5.10 (4.70, 5.54) | 5.10 (4.70, 5.53) | 5.26 (4.96, 5.95) | <0.001 |
| Serum uric acid (mg/dL) | 5.04 (4.10, 6.10) | 5.04 (4.10, 6.09) | 5.37 (4.54, 6.51) | <0.001 | 5.04 (4.10, 6.11) | 5.04 (4.10, 6.10) | 5.54 (4.71, 6.56) | <0.001 |
| Сreatinine (mg/dL) | 69 (62, 77) | 69 (62, 76) | 70 (63, 80) | 0.016 | 69 (62, 76) | 68 (62, 76) | 70 (64, 80) | 0.024 |
| HR (bpm) | 72 (66, 78) | 72 (66, 78) | 75 (67, 82) | 0.002 | 72 (66, 78) | 72 (66, 78) | 72 (66, 79) | 0.487 |
| DM |  |  |  | <0.001 |  |  |  | <0.001 |
| No | 12,517 (97%) | 12,239 (97%) | 278 (89%) |  | 5,369 (97%) | 5,250 (97%) | 119 (89%) |  |
| Yes | 402 (3.1%) | 368 (2.9%) | 34 (11%) |  | 166 (3.0%) | 152 (2.8%) | 14 (11%) |  |

^1^Quantitative data are expressed as median with interquartile range (IQR). Categorical data are presented as amounts with percentages.

^2^Comparisons of differences between groups are analyzed by Wilcoxon rank sum test for continuous variables and by Pearson’s Chi-squared test for categorical variables.

*Russian deprivation index measures general deprivation, and its components measure social, economic and environmental deprivation, respectively.

Q1 – the least deprived region; Q4 – the most deprived region

Q, quantile; CVD, Cardiovascular diseases; SBP, Systolic blood pressure; HDL-C, High-density lipoprotein cholesterol; LDL-C, Low-density lipid cholesterol; HR, Heart rate; BMI, Body mass index; TG, Triglycerides; DM, Diabetes mellitus.
