# Supplementary material for: Development and validation of nomograms including individual- and area-level variables to predict risk of fatal and non-fatal cardiovascular diseases among Russian population
Source: PLoS One. 2025 Jun 2;20(5):e0324736. doi: 10.1371/journal.pone.0324736 (PMC12129350; doi:10.1371/journal.pone.0324736)
Supplement: S9 Table — (DOCX) [file pone.0324736.s009.docx]

**S9 Table. Multivariate analysis of CVD-free survival in the training set.**

| Characteristic | Model 1* | | | Model 2 | | | Model 3 | | | Model 4 | | |
| --- | --- | --- | --- | --- | --- | --- | --- | --- | --- | --- | --- | --- |
|  | HR | 95% CI | p-value | HR | 95% CI | p-value | HR | 95% CI | p-value | HR | 95% CI | p-value |
| Serum uric acid | 0.96 | 0.84, 1.11 | 0.577 | 0.97 | 0.84, 1.11 | 0.615 | 0.96 | 0.83, 1.11 | 0.567 | 0.97 | 0.85, 1.11 | 0.678 |
| Smoking status |  |  |  |  |  |  |  |  |  |  |  |  |
| Current | Reference | — |  | — | — |  | — | — |  | — | — |  |
| Former | **0.71** | **0.55, 0.92** | **0.011** | **0.71** | **0.54, 0.91** | **0.008** | **0.72** | **0.55, 0.94** | **0.015** | **0.72** | **0.55, 0.95** | **0.022** |
| Never | **0.48** | **0.34, 0.67** | **<0.001** | **0.45** | **0.32, 0.65** | **<0.001** | **0.48** | **0.34, 0.67** | **<0.001** | **0.47** | **0.34, 0.65** | **<0.001** |
| Age | **1.07** | **1.05, 1.09** | **<0.001** | **1.07** | **1.05, 1.09** | **<0.001** | **1.07** | **1.05, 1.09** | **<0.001** | **1.07** | **1.05, 1.09** | **<0.001** |
| Sex |  |  |  |  |  |  |  |  |  |  |  |  |
| Men | Reference | — |  | — | — |  | — | — |  | — | — |  |
| Women | **0.57** | **0.36, 0.92** | **0.022** | **0.58** | **0.36, 0.93** | **0.023** | **0.56** | **0.35, 0.89** | **0.014** | **0.58** | **0.36, 0.93** | **0.024** |
| Сreatinine | 1.00 | 1.00, 1.01 | 0.306 | 1.00 | 1.00, 1.01 | 0.272 | 1.00 | 1.00, 1.01 | 0.413 | 1.00 | 1.00, 1.00 | 0.419 |
| Fasting glucose | 1.04 | 0.96, 1.13 | 0.323 | 1.05 | 0.97, 1.13 | 0.246 | 1.04 | 0.96, 1.12 | 0.373 | 1.04 | 0.97, 1.13 | 0.285 |
| SBP | **1.01** | **1.00, 1.01** | **0.007** | **1.01** | **1.00, 1.01** | **<0.001** | **1.01** | **1.00, 1.01** | **0.006** | **1.01** | **1.00, 1.01** | **0.003** |
| BMI | **1.03** | **1.01, 1.06** | **0.004** | **1.03** | **1.01, 1.06** | **0.003** | **1.03** | **1.01, 1.06** | **0.005** | **1.03** | **1.01, 1.05** | **0.003** |
| TG | 1.02 | 0.94, 1.12 | 0.624 | 1.02 | 0.93, 1.11 | 0.712 | 1.02 | 0.93, 1.12 | 0.672 | 1.02 | 0.93, 1.11 | 0.682 |
| LDL-C | **1.15** | **1.03, 1.29** | **0.014** | **1.18** | **1.06, 1.31** | **0.003** | **1.16** | **1.04, 1.28** | **0.005** | **1.16** | **1.05, 1.29** | **0.005** |
| HDL-C | **0.75** | **0.57, 0.97** | **0.032** | **0.74** | **0.56, 0.99** | **0.040** | **0.75** | **0.58, 0.98** | **0.038** | **0.74** | **0.56, 0.98** | **0.033** |
| DM |  |  |  |  |  |  |  |  |  |  |  |  |
| No | Reference | — |  | — | — |  | — | — |  | — | — |  |
| Yes | **1.65** | **1.08, 2.54** | **0.022** | **1.62** | **1.05, 2.49** | **0.029** | **1.71** | **1.13, 2.57** | **0.011** | **1.72** | **1.15, 2.57** | **0.008** |
| HR | 1.01 | 1.0, 1.02 | 0.217 | 1.01 | 0.99, 1.02 | 0.294 | 1.01 | 1.00, 1.02 | 0.116 | 1.01 | 0.99, 1.02 | 0.262 |
| General deprivation** |  |  |  |  |  |  |  |  |  |  |  |  |
| Q1 | Reference | — |  |  |  |  |  |  |  |  |  |  |
| Q2 | **1.19** | **1.01, 1.39** | **0.032** |  |  |  |  |  |  |  |  |  |
| Q3 | **1.56** | **1.04, 2.33** | **0.032** |  |  |  |  |  |  |  |  |  |
| Q4 | 1.02 | 0.83, 1.25 | 0.869 |  |  |  |  |  |  |  |  |  |
| Social deprivation |  |  |  |  |  |  |  |  |  |  |  |  |
| Q1 |  |  |  | Reference | — |  |  |  |  |  |  |  |
| Q2 |  |  |  | **1.75** | **1.21, 2.54** | **0.003** |  |  |  |  |  |  |
| Q3 |  |  |  | 1.00 | 0.83, 1.21 | 0.971 |  |  |  |  |  |  |
| Q4 |  |  |  | 0.99 | 0.74, 1.34 | 0.958 |  |  |  |  |  |  |
| Economic deprivation |  |  |  |  |  |  |  |  |  |  |  |  |
| Q1 |  |  |  |  |  |  | Reference | — |  |  |  |  |
| Q2 |  |  |  |  |  |  | 0.88 | 0.65, 1.19 | 0.418 |  |  |  |
| Q3 |  |  |  |  |  |  | 1.23 | 0.82, 1.83 | 0.317 |  |  |  |
| Q4 |  |  |  |  |  |  | 1.13 | 0.87, 1.47 | 0.361 |  |  |  |
| Environmental  deprivation |  |  |  |  |  |  |  |  |  |  |  |  |
| Q1 |  |  |  |  |  |  |  |  |  | Reference | — |  |
| Q2 |  |  |  |  |  |  |  |  |  | 1.02 | 0.91, 1.15 | 0.693 |
| Q3 |  |  |  |  |  |  |  |  |  | 1.15 | 1.00, 1.33 | 0.058 |
| Q4 |  |  |  |  |  |  |  |  |  | 1.31 | 0.91, 1.89 | 0.146 |

All models include the same laboratory and anthropometric indicators.

*Model 1 includes an indicator of general deprivation, model 2 – social deprivation, model 3 – economic deprivation, model 4 – environmental deprivation.

**Russian deprivation index measures general deprivation, and its components measure social, economic and environmental deprivation, respectively.

Q1 – the least deprived region; Q4 – the most deprived region

HR, Hazard Ratio; CI, Confidence Interval; Q, quantile; CVD, Cardiovascular diseases; SBP, Systolic blood pressure; HDL-C, High-density lipoprotein cholesterol; LDL-C, Low-density lipid cholesterol; HR, Heart rate; BMI, Body mass index; TG, Triglycerides; DM, Diabetes mellitus.
